# Chemistry and Pharmacology of *Citrus sinensis*

**DOI:** 10.3390/molecules21020247

**Published:** 2016-02-22

**Authors:** Juan Manuel J. Favela-Hernández, Omar González-Santiago, Mónica A. Ramírez-Cabrera, Patricia C. Esquivel-Ferriño, María del Rayo Camacho-Corona

**Affiliations:** 1 Facultad de Ciencia Químicas, Universidad Juárez del Estado de Durango, Av. Artículo 123 S/N, Núcleo Universitario, Col. Filadelfia, C.P. 35015, Gómez Palacio, Durango, Mexico; jackman610@ujed.com.mx; 2 Facultad de Ciencias Químicas, Universidad Autónoma de Nuevo León, Av. Universidad S/N Ciudad Universitaria, C.P. 66451, San Nicolás de los Garza, Nuevo León, Mexico; omar.gonzalezst@uanl.edu.mx (O.G.S.); monica.ramirezcbr@uanl.edu.mx (M.A.R.C.); patricia.esquivelfr@uanl.edu.mx (P.C.E.F.)

**Keywords:** *Citrus sinensis*, orange, chemistry, pharmacological activities, natural products

## Abstract

Presently the search for new drugs from natural resources is of growing interest to the pharmaceutical industry. Natural products have been the source of new drugs since ancient times. Plants are a good source of secondary metabolites which have been found to have beneficial properties. The present study is a review of the chemistry and pharmacology of *Citrus sinensis.* This review reveals the therapeutic potential of *C. sinensis* as a source of natural compounds with important activities that are beneficial for human health that could be used to develop new drugs.

## 1. Introduction

Natural products have been a rich source of compounds for drug discovery and offer larger scale structural diversity than synthetic compounds. Natural products have been major sources of bioactive agents and will continue to play a protagonist role in the discovery of new drugs [1]. The genus *Citrus* belongs to the family *Rutaceae*, This genus is the most important fruit tree crop in the world, with an annual production of approximately 123 million tons in 2010 [2,3]. Various species of *Citrus* are useful, such as *C. limon* (lemon), *C. medica* (citron), *C. aurantium* (sour orange), *C. paradisi* (grapefruit), *C. reticulata* (mandarin, tangerine), *C. clementina* (clementine) and *C. sinensis* (sweet orange) [4]. This review presents a botanical description of *C. sinensis*, its traditional uses, chemical composition, and pharmacological studies.

## 2. Botanical Description

*C. sinensis* represents the largest citrus cultivar groups grown around the world, accounting for about 70% of the total annual production of *Citrus* species [5]. *C. sinensis* is native to Asia and is now widespread throughout the Pacific and warm areas of the world. *C. sinensis* is an evergreen flowering tree. The height of orange trees is generally 9–10 m, with large spines on branches. Leaves are alternate, with narrowly winged-petioles (3–5 mm wide, 6.5–15 cm long); the shape of blades ranges from elliptical, oblong to oval, bluntly toothed and they emit a strong characteristic citrus odor due to the presence of copious oil [6]. Flowers are axillary borne singly or in whorls of 6 (5 cm wide) with five white petals and 20–25 yellow stamens. The fruit may be globose to oval (6.5 to 9.5 cm wide) and ripens to orange or yellow. Anatomically, the fruit consists of two distinct regions, the pericarp, also called the peel, skin or rind, and the endocarp or pulp with juice sac glands [7,8]. The skin consists of an epidermis of epicuticular wax with numerous small aromatic oil glands that give of its particular smell. The pericarp consists of the outer flavedo or epicarp, largely made of parenchymatous cells and cuticle [9,10]. The albedo or mesocarp lying beneath the flavedo consists of tubular-like cells joined together to constitute the tissue mass compressed into the intercellular area. The fruit usually contains a sweet pulp and several to numerous seeds within [11]. The fruit pulp is typically formed of eleven segments of juice filled with flavor that goes from sour to sweet. In orchards it is sensitive to frost. The fruit is perennial and it has adapted to a variety of climates [12].

## 3. Traditional Uses

*C. sinensis* is consumed all over the world as an excellent source of vitamin C, which is a powerful natural antioxidant that builds the body’s immune system [13]. It has been used traditionally to treat ailments like constipation, cramps, colic, diarrhea, bronchitis, tuberculosis, cough, cold, obesity, menstrual disorder, angina, hypertension, anxiety, depression and stress [14].

## 4. Chemical Composition

**Figure 1:**
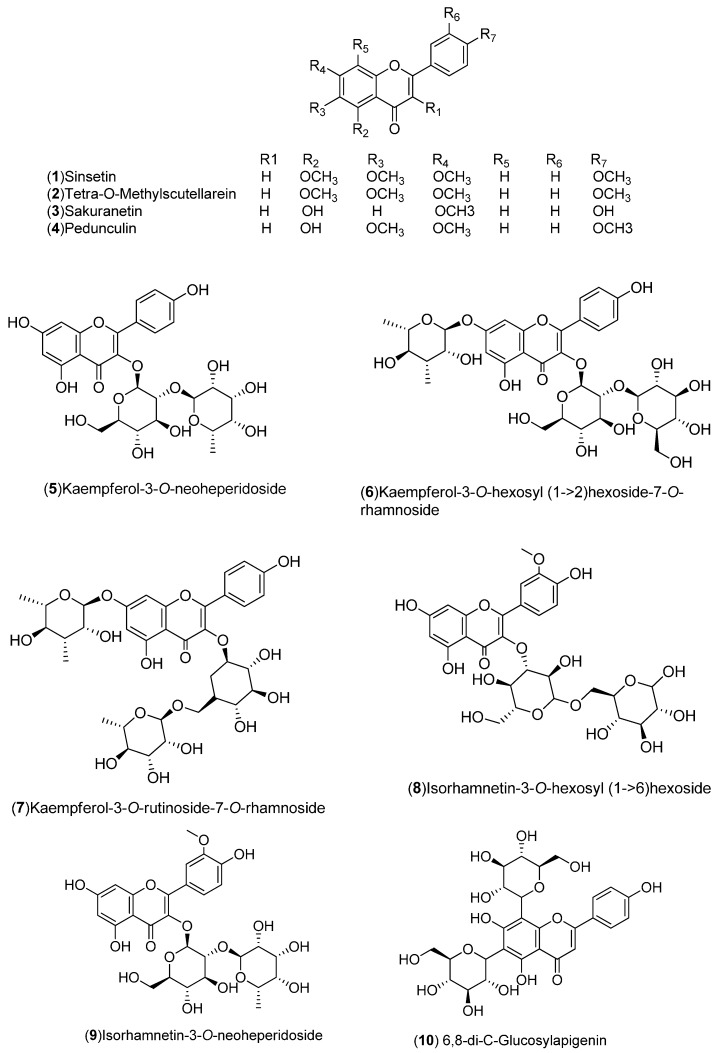

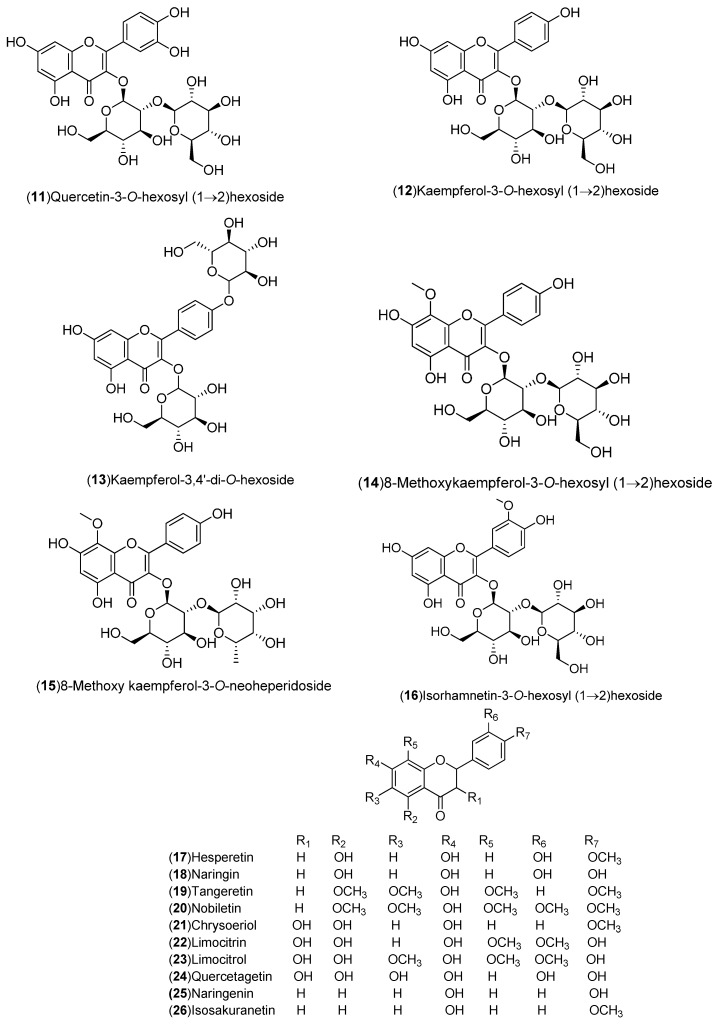

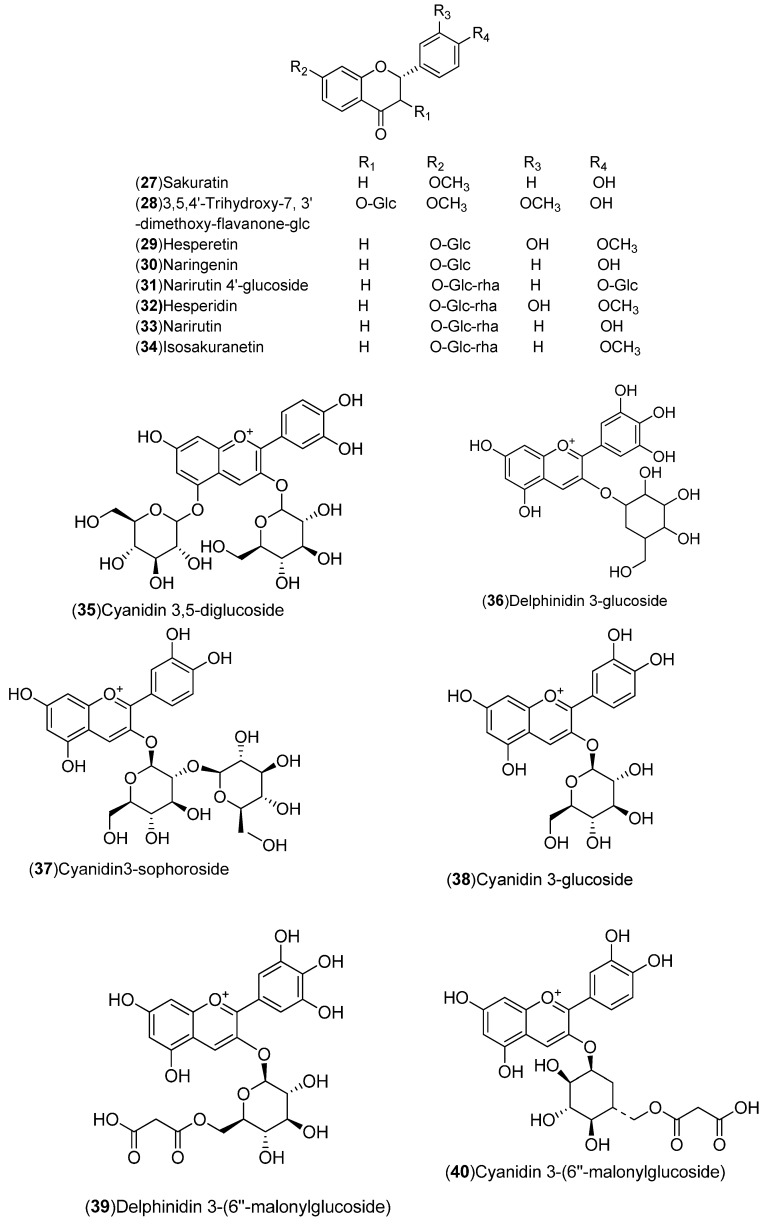

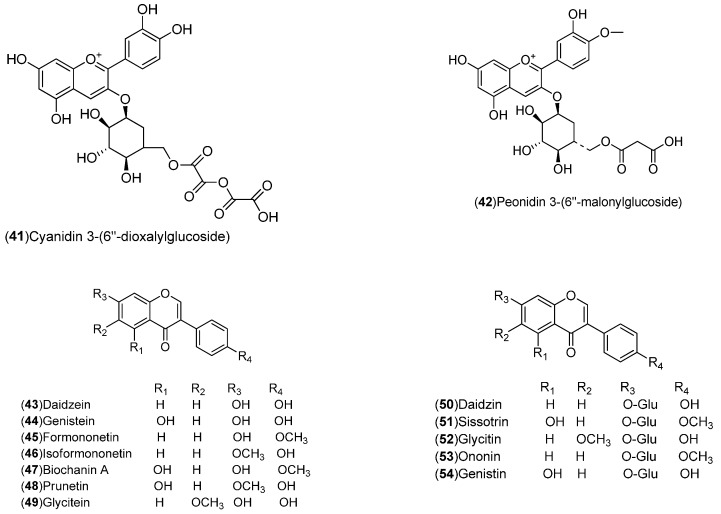
Chemical structures of flavonoids.

**Figure 2:**
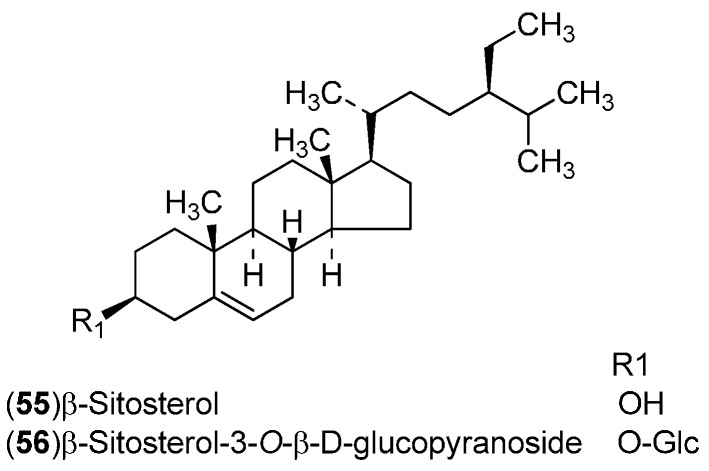
Chemical structures of steroids.

**Figure 3:**
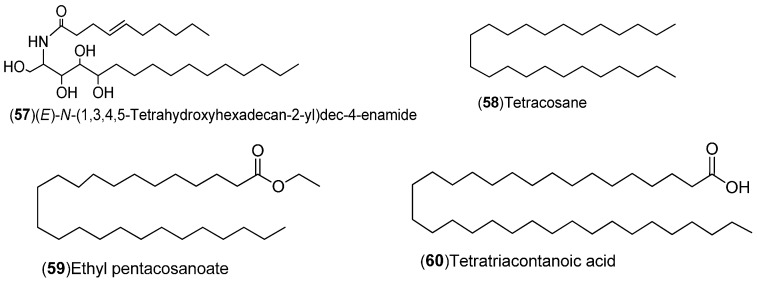
Chemical structures of hydroxyamides, alkanes and fatty acids.

**Figure 4:**
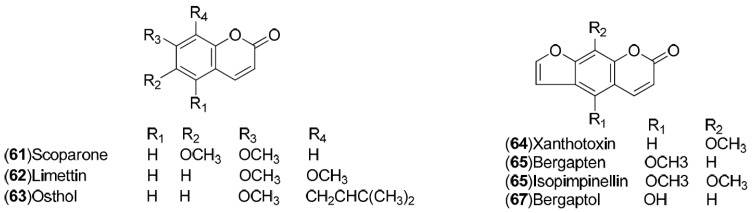
Chemical structures of coumarins.

**Figure 5:**
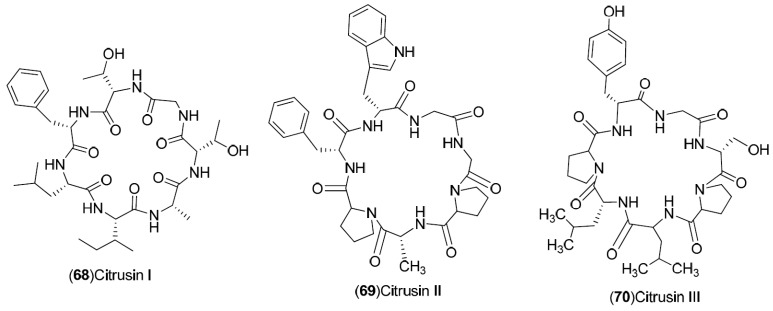
Chemical structures of peptides.

**Figure 6:**
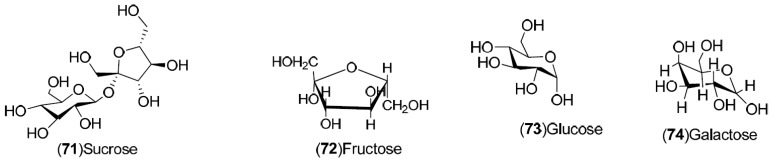
Chemical structures of carbohydrates.

**Figure 7:**
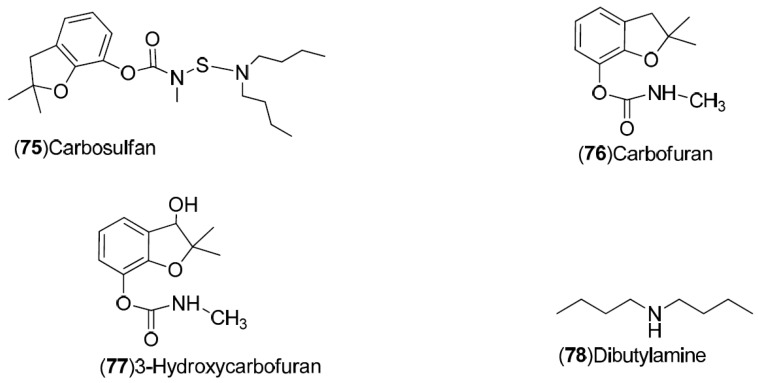
Chemical structures of carbamates and alkylamines.

**Figure 8:**
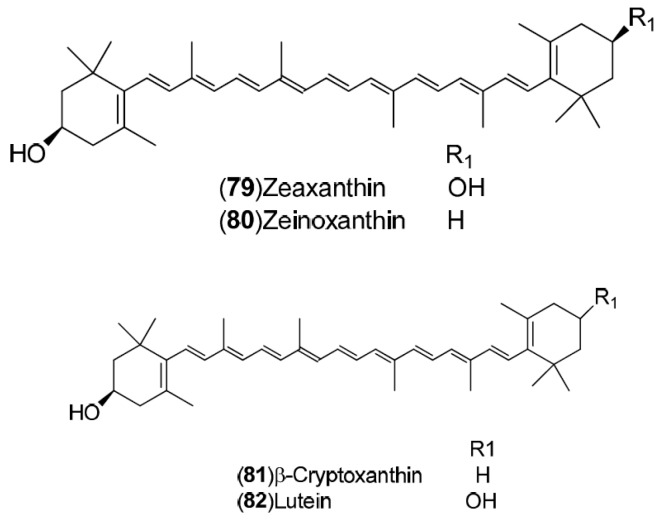
Chemical structures of carotenoids.

**Figure 9:**
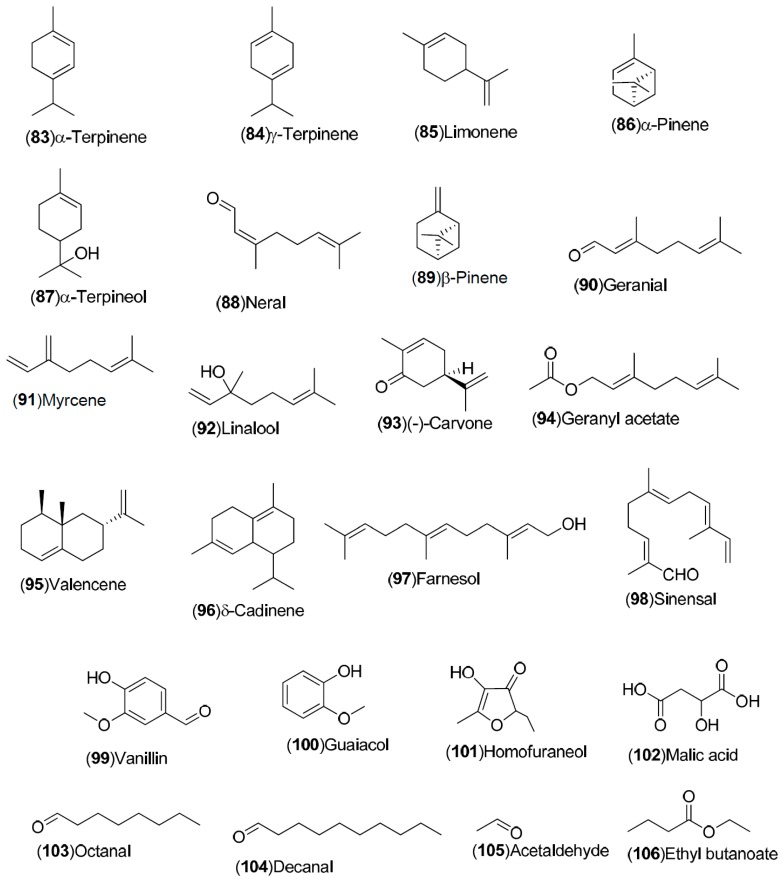

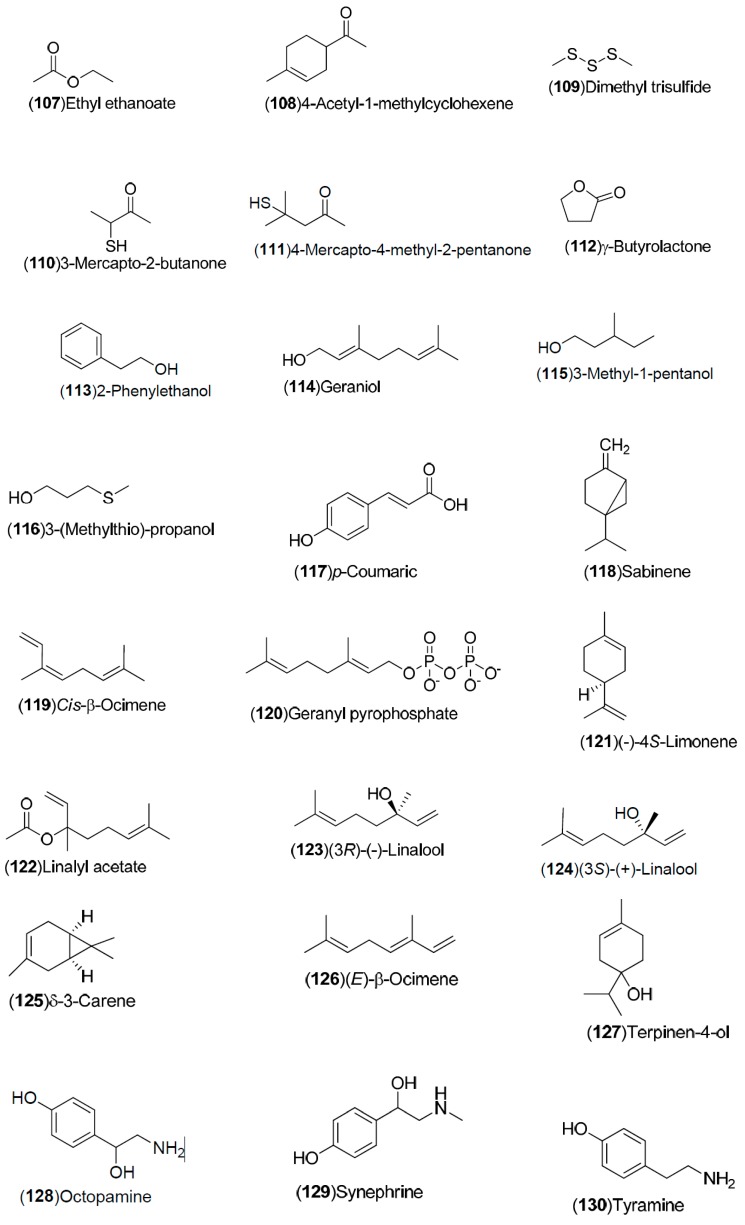

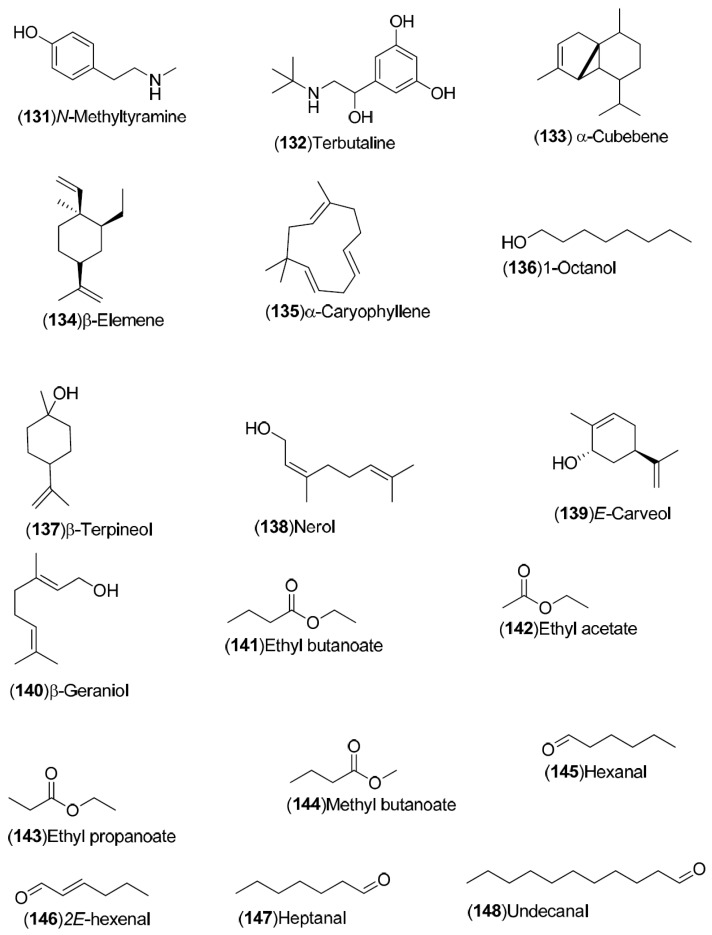
Chemical structures of volatile compounds.

*C. sinensis* is a rich source of secondary metabolites which contribute to the pharmacological activities attributed to this plant. Several types of chemical compounds have been identified in fruits, peel, leaves, juice and roots of *C. sinensis*, which include the following groups: flavonoids **1**–**54** [15,16,17,18,19,20,21,22,23,24,25]. steroids **55**,**56**, hydroxyamides, alkanes and fatty acids **57**–**60** [18], coumarins **61**–**67** [26,27,28,29], peptides **68**–**70** [30], carbohydrates **71**–**74** [31], carbamates and alkylamines **75**–**78** [32], carotenoids **79**–**82** [33], volatile compounds **83**–**148** [34,35,36,37,38,39], and nutritional elements such as potassium, magnesium, calcium and sodium [40]. Table 1 lists the different groups of compounds, region of collection, plant and references. The chemical structures of constituents isolated and characterized in *C. sinensis* are shown in Figure 1, Figure 2, Figure 3, Figure 4, Figure 5, Figure 6, Figure 7, Figure 8 and Figure 9.

The results of these studies clearly demonstrate that orange is a good source of compounds that could have good potential for incorporation into human food products as functional ingredients and for new drugs, as we shall see from their pharmacological activities.

## 5. Pharmacological Activities

### 5.1. Antibacterial Activity

The antibacterial activity of essential oil, crude extracts and pure compounds of *C. sinensis* has been demonstrated in several studies. Silver nanoparticles synthesized at 25 °C and 60 °C using *C. sinensis* peel aqueous extract, showed diverse zones of inhibition using the agar well-diffusion method against *Escherichia coli* (25 °C 12.5 mm, 60 °C 16.0 mm), *Pseudomonas aeruginosa* (25 °C 11.7 mm, 60 °C 13.4 mm) and *Staphylococcus aureus* (25 °C 7.8 mm, 60 °C 9.2) [41]. Another study showed that silver nanoparticles synthesized by mixing silver nitrate solution with *C. sinesis* juice for 2 h at 37 °C displayed minimum inhibitory concentration (MIC) values of 20 μg/mL for *Bacillus subtilis* and *Shigella* and 30 μg/mL for *S. aureus* and *E. coli*. Antibiofilm activity of 80% to 90% was observed at 25 μg/mL [42]. Cold-pressed terpeneless (CPT) *C. sinensis* oil dissolved in ethanol or dimethylsulphoxide (DMSO) displayed MICs for *Listeria monocytogenes* at 0.3% and 0.25% *v*/*v*, and for *Salmonella typhimurium* at 1% *v*/*v*. Both ethanol and DMSO oil dispersion systems exhibited an intermediate MIC of 0.75% *v*/*v* for *Lactobacillus plantarum* [43]. CPT at 0.5% *v*/*v* also displayed an inhibition zone effect at 10 µL against *S. aureus* strains: methicillin-susceptible strain (31.50 ± 3.02 mm); methicillin-resistant strain (65.83 ± 3.76 mm); methicillin- and vancomycin intermediate-resistant strains (76.67 ± 4.08 mm and 32.50 ± 2.74, respectively). Inhibition of bacterial growth in the plates containing test oil was judged by comparing with the visible growth of untreated control plates [44]. The essential oil containing 1,8-cineole and hydrocarbons showed MIC_90%_ ≥ 10% (*v*/*v*) against *P. aeruginosa* [45]. Another study showed that *C. sinensis* oil showed diverse inhibition diameters with 0.1 mL of the oil against *E. coli* (18 ± 2 mm), *L. monocytogenes* (27 ± 2 mm), *B. cereus* (19 ± 2 mm) and *S. aureus* (14 ± 3 mm) [46]. Sweet orange oil and its major compounds decanal (73.36%), octanal (78.12%), and linalool (90.61%) obtained by molecular distillation and column chromatography showed inhibitory bactericidal effects on *E. coli* (MIC 100–25 µg/mL; MBC 200–50 µg/mL), *S. aureus* (MIC 100–50 µg/mL; MBC 200–100 µg/mL), *Saccharomyces cerevisiae* (MIC 100–6.25 µg/mL; MBC 200–25 µg/mL), *Aspergillus niger* (MIC 50 µg/mL; MBC 200–100 µg/mL) and no activity for *Penicillium citrinum* [47]. A mixture (1:1 *v*/*v*) of *C. sinensis* and *C. bergamia* essential oils showed inhibitory activity with a MIC value of 0.25%–0.5% (*v*/*v*) and a minimum inhibitory dose (MID) of 50 mg/L against vancomycin-susceptible and vancomycin-resistant *Enterococcus faecium* and *E. faecalis*, respectively. The predominant components of the mixture were limonene (45%–73%), citral (0.7%–3%) and linalool (0.5%–15%) [48,49]. Terpene oil obtained from *C. sinensis* essence produced 29.2 ± 3.7 mm zones of inhibition with 10 µL of oil, against eleven strains/serotypes of *Salmonella* (*S. enteritidis*, *S. senftenberg*, *S. senftenberg*, *S. tennessee*, *S. kentucky*, *S*. *heidelberg*, *S. montevideo, S. michigan*, *S. typhimurium*, and *S. Stanley*). The most predominant compound in the oil was d-limonene at a level of about 94%. Myrcene was the second most predominant compound, accounting for about 3% of the oil [50]. *C. sinensis* oil had strong antibacterial activities on *L. monocytogenes*, *E. coli*, *S. enteritidis*, *P. mirabilis* and *B. cereus* [51]. An anti-acne formulation based on *C. sinensis* (3%), *Ocimum basilicum* L. (5%) essential oils and acetic acid (12%) inhibited *Propionibacterium acnes.* This antibacterial activity is due mainly to the 94.0% of limonene in *C. sinensis,* and limonene (2.54%), linalool (21.0%) and eugenol (7.17%) in *O*. *basilicum* L. [52]. Peel hexane extract of *C. sinensis* displayed antimycobacterial activity against drug-sensitive (MIC 200 µg/mL), isoniazid-resistant *(*MIC 25 µg/mL), and ethambutol-resistant (MIC 50 µg/mL) variants of *Mycobacterium tuberculosis* H37Rv. Streptomycin showed a MIC value of 0.50 µg/mL for the sensitive strain whereas a MIC >8 µg/mL for the resistant strain. Isoniazid displayed a MIC value of 0.60 µg/mL for the sensitive strain whereas for the resistant strain was >1 µg/mL. Ethambutol gave a MIC value of 2 µg/mL for the sensitive strain and for the resistant strain was >32 µg/mL. Finally, rifampicin showed a MIC value of 0.60 µg/mL for the sensitive strain and a MIC >2 µg/mL for the resistant strain. In this study the standard drugs displayed better activity than the tested extracts. However, from the most active extract it is possible to obtain compounds with better activity than standard drugs [53]. Acetone and hexane extracts of *C. sinensis* leaf showed inhibition zones of 27 mm towards *Helicobacter pylori.* Clarithromycin (0.05 μg/mL) was added as positive control, and this antibiotic displayed better activity than the tested extracts. It is important to point out that active extracts could contain compounds with better activity [54]. The above results clearly indicate the wide antibacterial spectrum of *C. sinensis* thus justifying its use as antibacterial agent.

### 5.2. Antifungal Activity

Antifungal activity of plant crude extracts, oils and secondary metabolites of *C. sinensis* has been reported. The compound 3-[4-hydroxy,3-(3-methyl-2-butenyl)-phenyl]-2-(*E*)-propenal isolated from hexane extract of injured peel of *C. sinensis* L. Osbeck cv*.* Valencia or *C. paradisa* MacFaden cv*.* Marsh showed activity against *Penicillium digitatum* and against *Cladosporium cucumerinum* on Si gel tlc plates using 7 µg of compound [55]. Aqueous, ethanol and petroleum ether extracts of *C. sinensis* L. (Osbeck) showed activity against *Candida albicans* [56]. Another study showed that an oil combination (1:1) of *C. maxima* Burm and *C. sinensis* L. (Osbeck) obtained by hydrodistillation caused 100% inhibition of the mycelial growth of *Aspergillus fumig*atus, *A. terreus*, *Alternaria alternate*, *Fusarium oxysporum*, *Helminthosporium oryzae*, and *Trichoderma viride* at 750 ppm [57]. Polymethoxylated flavones obtained from *C. sinensis* peel extract (flavone-7-*O*-[6-acyl]-glucoside, tetramethyl-O-scutellarein, nobiletin, natsudaidai, tangeretin, heptamethoxyflavone) showed activity against *Aspergillus niger* (MIC ≥ 1.6 mg/mL) using a microbroth dilution assay [58]. The hydrodistilled essential oils of six different varieties of *C. sinensis* showed antifungal efficacy against *P. digitatum* (ED_50_ 2389.9–1004.6 ppm) and *P. italicum* (ED_50_ 5407.5–3142.2 ppm). Essential oil from peels obtained by cold-pressing method showed activity against *Mucor hiemalis*, *P. expansum* and *F. proliferatum* having inhibition of 36.5%, 34.9% and 59.5% using the agar dilution technique [59]. The increasing worldwide incidence of fungal infections has created the need to search for new antifungal agents, and in this context *C. sinensis* offers a variety of compounds with antifungal activity.

### 5.3. Antiparasitic Activity

Parasitic diseases, are serious worldwide public health problems, and *C. sinensis* is an alternative in the treatment and control of these diseases. The hexane (IC_50_ 42.13 µg/mL), chloroform (IC_50_ 88.03 µg/mL), ethyl acetate (IC_50_ 26.67 µg/mL), acetone (IC_50_ > 100 µg/mL), and methanol (IC_50_ > 100 µg/mL) extracts of *C. sinensis* peel, displayed moderate antimalarial activity against chloroquine (CQ)-sensitive (3D7) strain of *Plasmodium falciparum.* In this study, various standard drugs were used: artemisinin (3D7 strain IC_50_ 0.0045 μg/mL), chloroquine (3D7 strain IC_50_ 0.021 μg/mL), CQ diphosphate (D6 strain IC_50_ 0.00311 μg/mL), mefloquine (D6 strain IC_50_ 0.01608 μg/mL) and quinine (3D7 strain IC_50_ 0.02 μg/mL) [60]. The petroleum ether and methanol extracts of C*. sinensis* showed moderate antimalarial activity against *P. falciparum* FCK 2 strain having IC_50_ values of 51.06 and 53.61 µg/mL, respectively. Untreated controls, consisting of parasitized red blood cells and 10 μL Ci of [^35^S]-methionine were used [61]. Essential oil of *C. sinensis* peel showed that a dose of 0.4 g/mL caused death of *Trypanosoma evansi* in 3 min, and induced death of *Trypanosoma brucei brucei* in 5 min. A set of positive control (25 mg/mL of diminavetor), negative control (infected blood suspended in heparin and Phosphate Buffer Saline Glucose pH 7.2) and diluent control (pure vegetable oil 100%) were set up. Diminavetor induced total lysis after 2 min for both parasites [62]. Although extracts showed weak antiparasitic activity, there is the need for the separation, purification and structural elucidation of pure compounds from extracts and essential oil of *C. sinensis* in order to find potential antiparasitic drugs.

### 5.4. Antiproliferative Activity

A standardized extract of red orange juice obtained from three pigmented varieties of *C. sinensis* (Moro, Tarocco, Sanguinello) inhibited proliferation of normal human prostatic epithelial cell line PZ-HPV-7 at 10^−3^ g/mL and lung fibroblast cell line of Chinese hamsters V79-4 at 10^−4^ g/mL. Untreated controls just with 5 × 104 cells/well were employed [63]. The juice of fruits of *C. sinensis* (L) Osb. (cv. Washington Navel and cv. Sanguinello) at concentrations of 82.6% and 73% showed 100% antiproliferative activity against the cell lines: K562 (human chronic myelogenous leukemia) and HL-60 (human leukemia). In the same way concentration of 10% showed 90.5% antiproliferative activity against MCF-7 cells (human breast adenocarcinoma) [64]. Another study showed the anti-proliferative and cytostatic effects of *C*. *sinensis* juice on the growth of guinea corn radicle has been documented. In this study it was shown that percentage of inhibition at concentrations of 5%, 10%, 20%, 40% and 60% (*v*/*v*) were of 18.94%, 72.37%, 91.96%, 99.72%, and 100% respectively. The concentrations of 40% and 60% (*v*/*v*) of juice showed cytostatic effects compared with the standard drug methotrexate (50 µg/mL) which showed 77.71% of inhibition [65]. Polymethoxyflavones isolated from peels of *C. sinesis* showed activity on human lung cancer cells. Nobiletin and 3,5,6,7,8,3′,4′-heptamethoxyflavone had a half inhibitory concentration (IC_50_) of 50 µM against H1299 cells, while 5-hydroxy-3,7,8,3′,4′-pentamethoxyflavone and 5-hydroxy-3,6,7,8,3′,4′-hexamethoxy-flavone showed IC_50_ values of 16.5 µM against H1299 cells. The above four flavonoids had similar activity towards human lung cancer cells H441 and H460 [66]. Cold-pressed orange peel oil containing a mixture of non-hydroxylated polymethoxyflavones (75.1%) and hydroxylated polymethoxyflavones (5.44%) and a mixture containing only hydroxylated polymethoxyflavones (97.2%) induce apoptosis in breast cancer cells MCF-7 with a Minimal Effective Concentration (ECmin) of 9.25 and 4.62 µg/mL, respectively [67]. Other study with Apc(Min/+) mice (a mouse model for human familial adenomatous polyposis) fed with 5% of orange peel extract containing 30% polymethoxyflavones (tangeretin 19.0%, heptamethoxyflavone 15.24%, tetramethoxyflavone 13.6%, nobiletin 12.49%, hexamethoxyflavone 11.06 and sinensitin 9.16%) decreased the development of tumors [68]. Several compounds also obtained from peel extract presented inhibitory activities against the proliferation of cells (IC_50_) and induced apoptosis (AC_50_) of HL-60 cell lines: 3,5,6,7,8,3′,4′-heptamethoxyflavone (IC_50_ 13.31 ± 1.28 µM; AC_50_ 33.88 ± 0.01 µM), nobiletin (IC_50_ 41.50 ± 7.01 µM; AC_50_ > 100 µM), 3,5,6,7,3′,4′-hexamethoxyflavone (IC_50_ 20.59 ± 1.01 µM; AC_50_ 92.10 ± 5.67 µM), 3′-hydroxy-5,6,7,8,4′-pentamethoxyflavone (IC_50_ 52.72 ± 0.22 µM; AC_50_ 94.62 ± 1.50 µM), 4′-hydroxy-5,6,7,8,3′-pentamethoxyflavone (IC_50_ 47.41 ± 3.64 µM; AC_50_ 87.10 ± 7.83 µM), 5-hydroxy-3,6,7,8,3′,4′-hexa-methoxyflavone (IC_50_ 4.16 ± 2.33 µM; AC_50_ 5.90 ± 0.11 µM), 5-hydroxy-3,6,7,3′,4′-pentamethoxyflavone (IC_50_ 2.07 ± 2.56 µM; AC_50_ 5.87 ± 0.13 µM) [69]. Another study demonstrated that D-limonene rich volatile oil obtained from blood oranges inhibited proliferation of colorectal cancer cells HT-29 at 1000 ppm [70]. Flavones and isoflavones showed inhibition of cell proliferation and induction of cell apoptosis on MCF-7 breast cancer cells: 5-hydroxy-3,6,7,8,3′,4′-hexamethoxyflavone (IC_50_ 2.50 μM; (ECmin) 1.56 μM), 5,6,7,4′-tetramethoxyflavone (IC_50_ 10.5 μM; ECmin 3.15 μM ), 3,5,6,7,8,3′,4′-heptamethoxyflavone (IC_50_ > 50 μM; ECmin 50 μM), 5,6,7,3′,4′-pentamethoxyflavone (IC_50_ > 50 μM; ECmin >50 μM). A Cell numbers (1 × 10^3^ cells) were presented as control [71]. The aqueous extract of *C. sinensis* L. (Osbeck) showed significant cytotoxic effect on cells of the Yoshida ascites sarcoma [72]. 4′-Geranyloxyferulic (0.141 ± 0.011 mg/g) obtained C. *sinensis* depicted a potential chemopreventive effect [73]. All of these antiproliferative features suggest that properties of extracts and pure compounds particularly flavonoids contained in *C. sinensis*, could be explored for chemopreventive and therapeutic purposes in cancer.

### 5.5. Antioxidant Activity

In recent years there has been increasing interest in plant antioxidants because of their potential health-promoting properties. The antioxidant activity of juices of *C. sinensis* acquired in a local supermarket based on kinetics of hydrogen peroxide scavenging displayed *k* values of 1.2 ± 0.3 ×10^3^ s^−1^ and 0.4 ± 0.1 ×10^3^ s^−1^. The significant variation in kinetic constants of H_2_O_2_ elimination in food samples points to its potential use as a relative indicator and control of antioxidant activity [74]. The total antioxidant activity of Moro *C. sinensis* crude juice was evaluated on the basis of its ability to scavenge 2,2-diphenyl-1-picrylhydrazyl (DPPH^•^), OH^•^ and 2,2′-azino-bis(3-ethylbenzothiazoline-6-sulphonic acid (ABTS^•+^) radicals and to reduce iron. It was found that Moro juice efficiently scavenge ABTS radical cations reaching up to 64% of quenching corresponding to 14.30 µM using Trolox equivalents (TE) as the reference antioxidant, it also was able to scavenge DPPH radicals with an antioxidant power corresponding to 14.39 ± 0.19 µM TE and eliminated about the 87% of hydroxyl radical generated at 16.40 µM TE. The above antioxidant activities are attributed to the presence of five *C*-glycosyl flavones: lucenin-2, vicenin-2, stellarin-2, lucenin-2-4′-methyl ether and scoparin; one 3-hydroxy-3-methylglutaryl glycosyl flavonol: 3-hydroxy-3-methylglutaryl glycosyl quercetin; and one flavone *O*-glycosides: chrysoeriol 7-*O*-neoesperidoside [75]. An amperometric biosensor for the quantification of the scavenging capacity of orange juices displayed values for natural orange juice at 1.01 mM of IC_50_/µM 10.6 ± 0.5, the one purchased commercially gave values at 2.27, 1.56, 0.91, 2.22 mM of IC_50_ values 17.0 ± 0.8 µM, 17.2 ± 0.7 µM, 26.4 ± 0.8 µM, 18.9 ± 0.9 µM, respectively, substances such as ascorbic acid (IC_50_ 30.3 ± 0.9 μM), caffeic acid (no significant results), gallic acid (no significant results), ferulic acid (no significant results), curcumin (no significant results), catechol (no significant results), quercetin (no significant results) were used for control and also for comparing. The orange juice showed the best activity [76]. Seed extract of *C. sinensis* exhibited antioxidant activity using reducing power and DPPH radical-scavenging assays, gallic acid (IC_50_ = 29.5 μM) was used as control standard [77]. The flavonoid content of several methanolic extract fractions of Navel orange peel (flavedo and albedo of *C. sinensis*) was first analysed phytochemically and then assessed for its antioxidant activity *in vitro*. The chemical structures of the fractionated constituents were originally determined by comparing their retention times and the obtained UV spectral data with the available bibliographic data and further verified by detailed LC-DAD-MS (ESI+) analysis. The main flavonoid groups found within the fractions examined were polymethoxylated flavones, *O*-glycosylated flavones, *C*-glycosylated flavones, *O*-glycosylated flavonols, *O*-glycosylated flavanones and phenolic acids along with their ester derivatives. In addition, the quantitative HPLC analysis confirmed that hesperidin is the major flavonoid glycoside found in the orange peel. The antioxidant activity of the orange peel methanolic extract fractions was evaluated by DPPH assay and the Co(II)/EDTA-induced luminol chemiluminescence assay. Results showed that orange peel methanolic extracts possess moderate antioxidant activity as compared with the activity observed for the aglycones, diosmetin (EC_50_ 71.79 ± 13.58 mg and hesperetin (EC_50_ 29.18 ± 2.80 7 mg). Quercetin (EC_50_ = 0.06 mg quercetin/mg DPPH) was used as positive control and the tested aglycones exhibited a significant higher hydroxyl scavenging activity than quercetin [78]. *C. sinensis* juice showed 84.81% DPPH antiradical effect at 100 μg/mL. In this study the ascorbic acid (96.36%) was used as positive control showing 96.36% DPPH antiradical effect [79]. The bound phenolic content of citrus peel showed DPPH scavenging at 1 mg/mL, OH scavenging at 4 mg/mL, Fe^2+^ chelating ability at 0.48 mg/mL and the inhibition of Fe^2+^ induced lipid peroxidation in pancreas at 142.8 μg/mL [80]. Acetone-water extract obtained from fresh edible part of red oranges fruits (*C. sinensis,* Torocco) displayed an intracellular antioxidant activity of 85% in Caco-2 cells at 50 mg/mL. Positive standard drugs—gallic acid and vitamin C—were used. The extract obtained from red oranges exhibited significant higher antioxidant activity than positive controls [81]. The antioxidant activity of methanol and ethanol extracts of *C. sinensis* peel showed a significant free radical scavenging activity generated by ABTS of 55.8% and 60.7%, respectively and DPPH scavenging activity based on its capability as hydrogen donator of 70% and 80%, respectively. Water (20–150 μL) and ascorbic acid were used as a control [82,83]. Dichloromethane extract and diethyl ether, ethyl acetate and *n*-butanol fractions obtained from methanolic extract showed scavenging activity expressed as EC_50_ ranging from 3 to 1.1 mg dry extract/mg DPPH. The ethyl acetate fraction showed the best antioxidant activity, which is due to the presence of *C*-glycosylated flavones, *O*-glycosylated flavones, polymethoxylated flavones, *O*-glycosylated flavanones and esters of phenolic acids. The positive controls, ascorbic acid, trolox and quercetin were used. Trolox was found to be 7.2 times more active, ascorbic acid was 9.1 times more active and quercetin 10.9 times more active than ethyl acetate fraction [84,85].The current state of knowledge shows the benefits of *C. sinensis* as antioxidant therapeutic agent. Thus we encourage the use of *C. sinensis* as a nutraceutical agent.

### 5.6. Hypocholesterolemic Activity

Literature data shows that *C. sinensis* possesses beneficial properties related with cholesterol which is a serious health problem. The administration of lyophilized *C. sinensis* juice at a dose of 5 g/kg in aqueous vehicle in a volume of 0.5 mL/100 g body weight for 15 days on adult male Wistar rats (200–250 g)*,* decreased plasma levels of cholesterol (31%), LDL (44%) and triglycerides (33%) [86]. Microsized insoluble fibers from *C. sinensis* fruits lowered the concentrations of serum triglycerides (15.6%–17.8%) and serum total cholesterol (15.7%–17.0%) by means of enhancing the excretion of cholesterol (123%–126%) and bile acids (129%–133%) in feces [87]. Studies of hypocholesterolemia activity of *C. sinensis* are few, however they are relevant and interesting, suggesting further research on this topic, which will be of great benefit, is warranted.

### 5.7. Anti-Obesity Activity

In the last years, several studies have recently evaluated the beneficial effects of *C. sinensis* and the active components in weight management and obesity. Moro juice extract (Morosil^®^, 400 mg/die) was able to induce a significant reduction in body mass index (BMI) after 4 weeks of treatment. Moreover, in subjects treated with Moro extract, body weight, BMI, waist and hip circumference were significantly different from the placebo group. It was suggested that the active compounds anthocyanins, hydroxycinnamic acids, flavone glycosides and ascorbic acid contained in Moro juice have a synergistic effect on fat reduction in humans [88]. The effects of citrange (*C. sinensis* × *Poncirus trifoliata*) fruit extracts in high-fat (HF) diet-induced obesity mice were studied. Female C57BL/6 mice were fed with a chow diet (control), an HF diet, HF diet supplemented with 1% *w*/*w* citrange peel extract (CPE) or 1% *w*/*w* citrange flesh and seed extract (CFSE) for 8 weeks. Results showed that both CPE and CFSE regulated the glucose metabolic disorders in obese mice. In CPE and CFSE-treated groups, the body weight, blood glucose, serum total cholesterol (TC) and low density lipoprotein cholesterol (LDL-c) levels were significantly reduced relative to those in the HF group. To explore the mechanisms of action of CPE and CFSE on the metabolism of glucose and lipid, related genes expressions in liver were assayed. In liver tissue, the expression level of peroxisome proliferator-activated receptor γ (PPARγ) and its target genes were down-regulated by CPE and CFSE as revealed by qPCR tests. In addition, both CPE and CFSE decreased the expression level of liver X receptor (LXR) α and β, both involved in lipid and glucose metabolism. Results suggested that CPE and CFSE administration could ameliorate obesity and metabolic disorders in HF diet-induced obesity mice probably through the inhibition of PPARγ and LXRs gene expressions. Among the main flavonoids of citrange extracts were naringin and poncirin both could be the bioactive compounds in this process [89]. Aqueous- methanol extracts of flavedo, albedo, and pulp of pooled samples of two varieties of Citrus fruits (*C. reticulate* and *C. sinensis*) efficiently prevented oxidative stress in human adipocytes with no cytotoxic effects [90]. Many medications have been used to manage obesity over the years. However, most of the anti-obesity drugs that were approved and marketed have now been withdrawn due to serious adverse effects, in this sense the results mentioned above reveal that the beneficial effects of citrus and the active components could be used in weight management and obesity.

### 5.8. Activity in Cardiovascular System

Varieties of C*itrus* are a rich source of dietary flavonoids which reduce the risk of adverse cardiovascular events. The drinking of commercial *C. sinensis* juice (CSJ) decreased diastolic and systolic blood pressure in 5.13% (*p* = 0.03) and −5.91% (*p* = 0.003) respectively in healthy volunteers using 500 mL/day of orange juice twice a day during four-weeks. However, the administration of natural CSJ during four weeks did not have significant effects on either diastolic or systolic blood pressure [91]. The water-ethanol and acetone leaf extracts of *C. sinensis* showed inotropic depression on the atria of guinea pigs of both sexes (300–500 g) having values of EC_50_ of 300 µg/mL and 140 µg/mL, respectively. Drugs control naloxone (10 μM), propranolol (1.5 μM atropine sulfate), atropine sulfate (1 μM propranolol), did not change the effect of the crude extract [92]. In fact, cardiovascular medications are indicated for and/or used to treat many different disorders, conditions and diseases affecting the cardiovascular system, but in some cases could produce intensified effects, toxic levels, side effects and/or adverse effects. The results of these investigations show that oranges can be used therapeutically to treat this disease and could be safe.

### 5.9. Antiosteoporotic Activity

Problems related with osteoporosis, characterized by a loss of bone mass, is a major health problem that affect persons of advanced age. Citrus showed a potential protective activity against osteoporosis. Administration of ethanol extract of leaves and peel of *C. sinensis* (5 mg/kg) on ovariectomized rats, increased trabecular bone mineral content and bone mineral density of tibia as well as improved the levels of phosphorus and calcium reducing the bone loss [93]. The effects of feeding orange pulp on bone quality in a male rat with osteoporosis model improved some characteristics of bone structure [94]. Thus, alternative approaches and investigations for managing osteoporosis are needed and in this regard some evaluations of the anti-osteoporotic activity of *C. sinensis*, showed that it could be beneficial, safe and effective in management of osteoporosis.

### 5.10. Protective of UV Activity

Excessive UV radiation produces genetic mutations that could develop skin cancer. Standardized extract prepared from red orange (Bionap^®^, 15 and 30 μg/mL) showed protective effects on ultraviolet B (shortwave) which induced damage in human keratinocytes. This activity could occur to the block of cellular oxidative stress-related events such as inflammation and apoptosis [95]. A supplement of red orange complex^®^ (*C. sinensis* varieties Moro, Tarocco and Sanguinello) produced by Bionap Company reduced UV-induced skin erythema on human healthy volunteers using a dose of 100 mg/daily for 15 days. Moreover, skin age spots pigmentation (melanin content) decreased from 27% to 7% when subjects were exposed to red orange extract [96]. The presented investigations demonstrate that *C. sinensis* protect cells from genotoxic effects of an UV radiation. Thus, orange can be a good candidate for sun protection products. However more research in this field is required.

### 5.11. Relaxant, Sedative and Anxiolytic Activities

Aromatherapy is the use of essential oils and extracts as an alternative treatment for medical purposes. Exposure to ambient odor of natural essential oil of *C. sinensis* showed a relaxant and sedative effect on dental patients [97]. The methanol and dichloromethane extracts obtained from the flowers of *C. sinensis* (L.) Osbeck showed a dose-dependent sedative effect in the exploratory cylinder model mice with an ED_50_ (ip) values of 47.04 ± 12.03 mg/kg and 129.15 ± 21.25 mg/kg, respectively. Hesperidin (ED_50_ = 11.34 ± 2.48 mg/kg) was identified in the methanol extract as the sedative active principle of this plant [98]. Anxiolytic activity of sweet orange aroma was demonstrated in forty male volunteers who were allocated to five different groups for the inhalation of sweet orange essential oil (test aroma: 2.5, 5, or 10 drops). Psychologic parameters (state-anxiety, subjective tension, tranquilization, and sedation) and physiologic parameters (heart rate and gastrocnemius electromyogram) were evaluated. Results gave scientific support to use as a tranquilizer by aromatherapists [99]. Sweet orange aroma (*C. sinensis* oil) in Wistar rats demonstrated anxiolytic-like activity. The animals were exposed to the orange aroma (100, 200 or 400 μL) for 5 min within a plexiglass chamber and were then immediately submitted to the behavior tests. All doses of *C. sinensis* oil showed anxiolytic effect. Diazepam (2 mg/kg) was used as positive control. *C. sinensis* almost showed the same effect as diazepam [100]. Because of the well-recognized therapeutic potentials of extract and compounds of *C. sinensis* to present relaxant and sedative-hypnotic activities, orange represents an excellent natural alternative.

### 5.12. Insecticidal Activity

Essential oil extracted from leaves of *C. sinensis* have insecticidal activity against larvae of *Culex pipiens molestus* (LC_50_ 60 mg/L), two main compounds terpineol and 1,8-cineole were the most effective against *C. pipiens molestus* bites offering complete protection during 1.6 and 2 h, respectively [101]. Essential oil of *C. sinensis* obtained from fruit peel presented activity against *Musca domestica* L. (LC_50_ 3.9 mg/dm^3^). Dimethyl 2,2-dichlorovinyl phosphate (LC_50_ of 0.5 mg/dm^3^), a volatile organophosphate was used as a positive control. The positive control showed better activity than essential oil [102]. Orange essential oil (LC_50_ 3.9 (1.2–13) mg/dm3 (95% CI)) with (+)-limonene as the main component kill *M. domestica L*. within 15 min or less. Deltamethrin (LC_50_ 9.2 (2.8–29.5) mg/dm3 (95% CI)) was used as positive control, and this was more effective than essential oil [103]. *C. sinensis* essential oil showed activity against larvae of *M. domestica* with lethal concentrations between 3.93 and 0.71 μL/cm^2^, while lethal time LT_50_ varied between 5.8 to 2.3 days [104]. Essential oil of fruit peels and seeds of *C. sinensis* killed the larvae and adults of *Triboluim castaneum* by contact action. Larval toxicity was found at 42.48 μL, 41.58 μL and 40.28 μL at 24, 48 and 72 h exposure. The adult toxicity was 45.46 μL, 53.28 μL and 44.55 μL at 24, 48, and 72 h exposure, respectively [105]. Volatile extract of *C. sinensis* peels showed insecticidal potency during 30 and 60 min, against certain number of mosquitos (41 ± 8; 78 ± 5), houseflies (22 ± 4; 72 ± 7) and cockroaches (31 ± 4; 85 ± 5) [106]. Essential oil of *C. sinensis* was tested against *Planococcus ficus* a mealybug pest in grape vine growing areas worldwide. The LC_50_ and LC_90_ in adults were 5.4 and 16.2 mg/mL, respectively. Whereas pre-ovipositing adult females LC_50_ and LC_90_ were 5.4 and 13.5 mg/mL, respectively [107]. *C. sinensis* oil was tested on the cowpea adult bruchid, *Callosobruchus maculatus* being toxic at 24 h exposure (LC_50_ = 269 µL/L) [108]. Peel extracts of *C. sinensis* showed larvicidal and nymphicidal activity. Chloroform extract showed activity against the larvae of *Anopheles subpictus* (LC_50_ = 58.25 ppm and LC_90_ = 298.31 ppm), the methanol extract was active against larvae of *Culex tritaeniorhynchus* (LC_50_ = 38.15 ppm and LC_90_ = 184.67 ppm) and the hexane extract was active against the nymph of *Aphis gossypii* (LC_50_ = 162.89 ppm and LC_90_ = 595.40 ppm) [109]. Ethanol extract of *C. sinensis* peels showed larvicidal and pupicidal activities against mosquito of *Anopheles stephensi* (LC_50_ 182.24–490.84 ppm); *Aedes aegypti* (LC_50_ 92.27–497.41 ppm); and *Culex quinquefasciatus* (LC_50_ 244.70–530.97 ppm). The ethanol extract of *C. sinensis* showed 100% repellency in 150 min and showed complete protection in 90 min at 350 ppm against *A. stephensi*, *A. aegypti* and *C. quinquefasciatus*. The adult mortality was found with ethanol extract of *C. sinensis* against *A. stephensi* (LC_50_ 272.19 ppm; LC_90_ 457.14 ppm), *A. aegypti* (LC_50_ 289.62 ppm; LC_90_ 494.88 ppm), and *C. quinquefasciatus* (LC_50_ 320.38 ppm; LC_90_ 524.57 ppm) [110]. *C. sinensis* oil was effective against *C. quinquefasciatus* larvae (LC_50_ 11 µg) [111]. *C. sinensis* oil had insecticidal activity against *A. aegypti*, *C. quinquefasciatus* and *A. dirus* [112]. *C. sinensis* oil presented fumigant toxicity against *Tetranychus urticae* with LC_50_ 2.22 µL/L. In this study eugenol and phosphine were used as positive controls having LC_50_ = 0.004 µL/L air and 100% mortality at 2 × 10^−3^ g/L [113].

*C. sinensis* var. pear peel essential oil showed insecticidal activity against *Bemisia tabaci* at 8.5 µL/L which caused 97% mortality and LC_50_ 3.80 µL/L of air. Eugenol (LC_50_ 0.20 µ/L/L of air) was used as a positive control. Eugenol showed better fumigant action than essential oil [114]. *C. sinensis* essential oil showed strong toxic effect on eggs of *Hyalomma dromedarii,* especially in earlier embryonic development at concentration of 1:20 (oil: ethanol 95% *v*/*v*) [115]. The hexane extract of *C. sinensis* leaf possessed moderate larvicidal efficiency against dengue vector. The bioassays resulted in an LC_50_ and LC_90_ values of 446.84 and 1370.96 ppm, respectively after 24 h of exposure [116]. Coumarins xanthyletin and seselin, and the limonoid limonin isolated from hexane extract of *C. sinensis* leaves displayed *in vitro* activity on the growth of *Xylella fastidiosa* at concentrations ranging from 1.00 to 2.00 mg/mL. A blank experiment was performed with DMSO/H_2_O (1:1) alone (100 μL of bacterial culture, 33.5 μL of DMSO and 33.5 μL of H_2_O) in the well and was used as a positive control [117]. Extract of *C. sinensis* displayed strong bioactivity against *Semia phis heraclei, Aphis craccivora, Tetranychus viennensis* and *T. trancatus* [118]. The results of the experiments showed that natural products of *C. sinensis* clearly affect the growth of diverse plagues, so orange has the potential for use as alternative crop protectants against most likely pest species.

It is important to point out that literature includes three reviews of *C. sinensis*, one concerning the utilization of its nutrients to make multiple products of added value such as essential oil, pectin, yellow pigments, feed and enzymes [119]. The second review reported the chemical constituents of *C. sinensis* varieties [120], and the third is about the chemical composition of the essential oil of sweet orange [121]. Our review reported the chemical composition and pharmacological activities of *C. sinensis* varieties and the potential use of this plant as a source of bioactive compounds.

## 6. Conclusions

Natural products have been and will be important sources of new pharmaceutical compounds. Recently, there has been a renewed interest in natural product research due to the failure of alternative drug discovery methods to deliver many lead compounds in key therapeutic areas. In this sense, considering the health benefits of *C. sinensis* it presents excellent options for treating or helping in a disease due to its bioactive compounds (drug candidates) that show important activities or for developing new products, there is the need for public enlightenment on the importance of *C. sinensis* and finding and discovering new and effective drug compounds, so this review represents an excellent source of information about this natural product.

## Figures and Tables

**Table 1:** Groups of compounds, region of collection and plant organ.

Compound	Region of Collection	Plant Organ	References
Flavonoids: **1**–**54**	United States: Washington, Florida (**2**, **3**, **21**, **27**, **50**–**54**). India: Hisar, Shahjahanpur (**1**, **4**). Pakistan (**19**, **28**). Italy: Sicily, Messina (**17**, **18**, **20**, **22**–**26**, **29**–**34**). Spain: Murcia, Huelva (**5**–**16**). Germany: Braunschweig (**35**–**42**). Czech Republic: Prague (**43**–**49**)	Peel, flavedo, molasses, whole fruit, leaves	[15,16,17,18,19,20,21,22,23,24,25]
Steroids: **55**–**56**	United States: Washington (**55**–**56**)	Leaves	[18]
Hydroxylamide, alkane, Fatty acids: **57**–**60**	United States: Washington (**57**–**60**)	Leaves	[18]
Coumarins: **61**–**67**	India: Shahjahanpur (**61**) United States: Florida (Lakeland) (**62**–**67**)	Peel, root	[26,27,28,29]
Peptides: **68**–**70**	Japan: Wakayama (**68**–**70**)	Peel	[30]
Carbohydrates: **71**–**74**	Sweden: Stockholm (**71**–**74**)	Fruit	[31]
Carbamates, alkylamines: **75**–**78**	Spain: Valencia (**75**–**78**)	Fruit	[32]
Carotenoids: **79**–**82**	Germany: Stuttgart (**79**–**82**)	Fruit	[33]
Volatile compounds: 83–148	Spain: Huelva (**83**–**85**, **87**, **88**, **90**, **121**–**124**, **138**–**141**). China: Songzi (Hubei) (**137**, **125**, **126**, **143**–**148**). Turkey: Dortyol–Hatay, Kozan (**98**–**101**, **105**–**110**, **116**, **120**). United States: Florida (**92**–**97**, **111**–**119**, **127**–**131**). Germany: Steinheim (**86**, **89**, **91**, **102**–**104**, **132**–**136**, **142**)	Fruit, orange blossom, peel, leave	[34,35,36,37,38,39]
Potassium, magnesium, calcium and sodium	China: Beijing	Natural and commercial juices	[40]
